# V2 vasopressin receptor mutations: future personalized therapy based on individual molecular biology

**DOI:** 10.3389/fendo.2023.1173601

**Published:** 2023-05-24

**Authors:** László Sándor Erdélyi, László Hunyady, András Balla

**Affiliations:** ^1^Department of Anesthesiology and Intensive Therapy, Semmelweis University, Budapest, Hungary; ^2^Department of Physiology, Faculty of Medicine, Semmelweis University, Budapest, Hungary; ^3^Institute of Enzymology, Research Center for Natural Sciences, Hungarian Academy of Sciences, Budapest, Hungary; ^4^ELKH-SE Laboratory of Molecular Physiology, Eötvös Loránd Research Network, Budapest, Hungary

**Keywords:** AVPR2 gene, G protein-coupled receptor (GPCR), nephrogenic diabetes insipidus (NDI), nephrogenic syndrome of inappropriate antidiuresis (NSIAD), type 2 vasopressin receptor (V2R), arginine-vasopressin (AVP)

## Abstract

The diluting and concentrating function of the kidney plays a crucial role in regulating the water homeostasis of the body. This function is regulated by the antidiuretic hormone, arginine vasopressin through the type 2 vasopressin receptor (V2R), allowing the body to adapt to periods of water load or water restriction. Loss-of-function mutations of the V2R cause X-linked nephrogenic diabetes insipidus (XNDI), which is characterized by polyuria, polydipsia, and hyposthenuria. Gain-of-function mutations of the V2R lead to nephrogenic syndrome of inappropriate antidiuresis disease (NSIAD), which results in hyponatremia. Various mechanisms may be responsible for the impaired receptor functions, and this review provides an overview of recent findings about the potential therapeutic interventions in the light of the current experimental data.

## 1 Introduction

The type 2 vasopressin receptor (V2R) plays an essential role in the regulation of body water homeostasis. As a G protein-coupled receptor (GPCR), the V2R transmits the hormonal signal of arginine-vasopressin (AVP) by activating heterotrimeric G proteins (1). The GPCR superfamily consists highly conserved proteins throughout the evolution and the disease-causing mutation of the V2R was one of the earliest discovered loss-of-function mutation (2). The medical significance of the GPCRs is reflected by the fact that at least 40% of modern therapeutical drugs affect these receptors or GPCR signaling cascades directly or indirectly (3). Therefore, GPCRs are of remarkable importance to the pharmacological industry. Chronic diseases, such as hypertension, heart failure, and obstructive pulmonary diseases affect sooner or later a significant fraction of the world population, creating a challenge for market-based research to solve these therapeutic problems. However, the prevalence of GPCR signaling related congenital diseases is orders of magnitude less, creating an absolute need for academic research to find clinical solutions for these patients. More than 600 loss-of-function mutations and over 100 gain-of-function mutations in GPCRs are responsible for numerous diseases. The fast progress of molecular biological diagnostics in the clinical medicine keep increasing the incidence of these rare diseases every day (1, 4). Research targeting these GPCR mutations has reciprocal effect: on one hand, it helps to form therapeutical strategies and find pharmacological solutions for these patients, but on the other hand these investigations also open opportunities to better understand the function and structure of GPCRs (5). This review focuses on the practical and hypothetical and hopefully future causal therapeutic strategies for the loss-of-function and gain-of function mutations of the V2R causing X-linked nephrogenic diabetes insipidus (XNDI) and nephrogenic syndrome of inappropriate antidiuresis (NSIAD), respectively (6, 7). Only supportive therapy may be implemented for these patients in a clinical setting (8). Understanding the individual molecular consequences of these mutations creates possibilities for personalized therapy.

## 2 Physiology of the V2R

The kidney plays an essential role in water and electrolyte homeostasis, regulating body water volume by adjusting salt excretion and diluting or concentrating urine. Not only thirst and water intake, but also water excretion are tightly regulated for proper water balance and plasma osmolality (9). According to the basic logic of renal function, the vast majority of the large volume (ca. 180 L/day) glomerular filtrate (water and electrolytes) is reabsorbed along the nephron (10). In healthy humans approximately 85% of the glomerular filtered fluid is reabsorbed in the proximal tubule and in the loop of Henle, and only 8-14.5% of the filtered fluid is reabsorbed in the collecting ducts. This latter is under tight hormonal control *via* vasopressin (arginine-vasopressin in humans, AVP; also called as antidiuretic hormone, ADH) and atrial natriuretic peptide (ANP) actions (11–14).

Hypovolemic states of the body (afferent signal from baroreceptors and volume receptors) or plasma osmolality change leads to increased AVP release from the posterior pituitary gland (15, 16). The released AVP hormone has pleiotropic regulatory effects on distant target tissues and organs including urine concentration, vasomotor tone regulation, thrombocyte aggregation and coagulation factor release, carbohydrate metabolism regulation, hormone release regulation, all of which are mediated by V1 and V2 receptors, that are part of the GPCR family. The effect on urine concentration in the kidney is mediated by the V2R, which is primarily located in the basolateral membranes of the collecting duct cells (17).

Binding of circulating AVP to the V2R activates several signaling cascades in the collecting duct cells: as a GPCR, binding of the natural agonist leads to Gs coupling and consequential increase in cytoplasmic 3’,5’-cyclic adenosine monophosphate (cAMP) concentration. The elevated level of cAMP has several effects, including increased protein kinase A (PKA) activity and initiation of Ca^2+^ oscillations depending on Epac (exchange protein directly activated by cAMP) activity (18, 19). As with the majority of GPCRs, the V2R activates a pleiotropic spectrum of pathways rather a signal transduction pattern. Some of these cascades are independent of cAMP, including protein kinase B, phosphatidylinositol kinase and ERK1/2 activation (20, 21). As it has been observed in V2R pathology, termination of signaling is equally important to its initiation. Binding of β-arrestin to the ligand-bound receptor uncouples the heterotrimeric G proteins from the activated receptors, leading to signal termination and internalization of the V2R (22).

The water permeability is regulated through the translocation of aquaporin-2 (AQP2) channels to the luminal membrane of the collecting duct principal cells. Binding of the AVP hormone to the V2R leads to the alteration of cellular distribution of AQP2 in a cAMP dependent way. PKA dependent phosphorylation of specific serine and threonine sites in AQP2 water channels initiates their translocation from intracellular vesicles to the plasma membrane and causes retention of AQP2 by reducing its internalization (11, 23). A V2R dependent long-term effect of AVP is to increase the expression of AQP2 in these cells. Both the cAMP-PKA-CREB (cAMP-response element binding protein) and Epac pathways enhance transcription, while Epac dependent Ca^2+^ oscillation are fundamental to the short-term effect on channel translocation (19, 24, 25). The AVP also regulates the urea permeability of the medullary collecting duct principal cells in a V2R dependent manner, enhancing the urea recycling and *via* this mechanism contributes to the buildup of corticopapillary osmotic gradient (26).

## 3 Pathophysiology of the V2R

### 3.1 Loss-of-function mutations

All of the mechanisms described above ensure the precise regulation of the renal function. Congenital mutations in the V2R have demonstrated its key role in water balance and electrolyte homeostasis. Loss-of-function, inactivating mutations of the V2R lead to nephrogenic diabetes insipidus (NDI), a disease, which is characterized by extreme polyuria. These mutations are responsible for 90% of all NDI cases, while the other 10% involve mutations in the AQP2 (27, 28). The X-linked NDI nomenclature (XNDI) reflects that the V2R coding gene (*AVPR2*) is located on the X-chromosome (in contrast to the AQP2, which is found on the 12^th^ autosomal chromosome). Although mostly males are diagnosed with XNDI, heterozygous females can also exhibit the symptoms of the NDI (29). The molecular basis for female XNDI is skewed X-inactivation leading to varying penetrance (proportion of individuals presenting symptoms of the carried mutation). The mechanism of X-inactivation compensates the additional gene amount (compered to hemizygote males) in females, leading to inactivation of one of the X-linked alleles. Statistically, the probability of biased (skewed) normal allele inactivation is low. Hence, most of the female carriers are asymptomatic (29). According to a study, 25% of heterozygous women carrying a mutant *AVPR2* gene had symptoms, and 6% had complete XNDI (30). Mild, subclinical XNDI in women is often diagnosed only after the birth of a male descendant showing marked symptoms (31). This phenomenon raises the possibility that XNDI has greater prevalence in the population than is currently recognized in the literature. Over the years, several excellent reviews have discussed the genetic background of XNDI, and the expanding number of loss-of-function mutations recognized in the *AVPR2* has reached around 270 in more than 300 families. Two-thirds of these mutations are missense mutations, which result in a single amino acid substitution in the protein (27, 32). Missense mutations carry the hope that causal therapy can be identified in the future, unlike other forms of mutation, such as large insertions, deletions, resulting in different proteins than the wild type receptor.

Functional characterization of a missense mutation involves the investigation of the pathophysiological consequences of the mutant V2R at the cellular level. Although sequencing analysis of the mutation in the *AVPR2* does not necessarily explain the failure in receptor function, a recent study has demonstrated that *in silico* computational approaches, long-term molecular dynamics and structural analysis can be useful in the prediction of the effect of the mutation on impaired vasopressin receptor function (33). Based on the cellular function and mechanism, mutations in the V2R can be classified, similarly to the classification of mutations causing cystic fibrosis. As we elaborate later in this review, this method of classification not only helps to understand the molecular biology of the V2R, but also predicts the possible causal therapeutic strategy - if there is any - for the mutation. Causal therapy targets directly the underlying cause and pathomechanism of a disease, usually leading to superior therapeutical results compared to supportive therapy.

Class I mutations are characterized by the lack of effective protein synthesis. Several mechanisms may be responsible for the cellular absence of the V2R, including mutations altering the promoter or splicing regions, or affecting mRNA stability. Mutations leading to premature synthesis terminations, such as frameshift and nonsense mutations (resulting in early stop codons), have dual consequences: they produce short products and suffer rapid degradation at the same time (28).

Class II mutations are one of the most extensively investigated mutations. The majority of *AVPR2* mutations belong to class II, thus more than 50% of all XNDI cases have similar pathomechanism (34). These mutations lead to full-length protein products (or almost full length through late stop codons), but the expressed mutant V2R molecules have folding insufficiency resulting in endoplasmic reticulum (ER) retention due to recognition by the quality control system of the cells and the retained receptors are degraded in the proteasomes (28). Maturing *de novo* synthetized proteins may also be retained in the Golgi (35). The mutations leading to intracellular retention of the misfolded receptors may result in otherwise functional V2Rs with greatly decreased cell surface expression (31, 32, 36).

Class III mutant receptors are synthesized as full-length proteins and reach the plasma membrane after the maturation process. However, despite the adequate cell surface expression, these mutant V2Rs have impaired receptor function. The class IIIa mutations result in reduced G protein binding and/or G protein activation ability, making them unable to generate effective signaling. The class IIIb mutant receptors also lead to impaired second messenger generation because the AVP binding affinity is decreased compared to the wild-type V2R (27, 28). The distribution of mutations resulting in class III functional impairment shares topological similarity: the class IIIa mutations often occur in the intracellular and transmembrane regions, whereas the class IIIb mutations usually occur in the extracellular and transmembrane regions of the receptor (28). Mutations affecting the ligand binding pocket of the receptor lead to class IIIb functional phenomenon, although mutations in other receptor regions may also lead to decreased ligand affinity (5).

Class IV mutation leads to inappropriate intracellular trafficking due to constitutive β-arrestin binding and internalization into endocytotic vesicles from the cell surface leading to decreased sensitivity to AVP stimulation (37). The R137H-V2R mutant (nomenclature reflecting the amino acid substitution and localization) is expressed on the cell surface and is able to activate G proteins, but in the absence of the natural ligand AVP, this receptor is constitutively phosphorylated despite the inactive G proteins (38, 39).

### 3.2 Gain-of-function mutations

Gain-of-function mutations of the V2R result in increased receptor dependent signal generation in collecting duct cells causing NSIAD. This disease was first described in 2005 as a congenital form of SIADH (syndrome of inappropriate antidiuretic hormone secretion) (7). Recently, another pathogenesis was proved to cause NSIAD: gain-of-function mutation of Gs gene (named *GNAS*) that makes a similar endocrine syndrome with additional symptoms (40). To date, only five mutations in the V2R have been discovered that lead to NSIAD. All these known mutations in the V2R lead to constitutive active conformation and consequential increased intracellular cAMP concentration even in the absence of the natural agonist AVP. Besides of basal cAMP generation, these mutants have distinctive characteristics. The R137C and R137L mutants were insensitive to V2R inverse agonist tolvaptan. Furthermore, constitutive basal activity could not be enhanced with AVP (39, 41). In contrast, F229V, I130N and L312S mutations showed not just only elevated basal cAMP concentration due to constitutive activity, but the cAMP signal could also be further increased upon AVP stimulus. More importantly, from a clinical perspective, these mutant V2Rs were sensitive to tolvaptan (42–44).

Constitutively active V2R mutations may also be classified in terms of β-arrestin binding and internalization properties. All of the inverse agonist sensitive mutants lacked basal β-arrestin2 binding despite their constitutive activity. F229V, I130N and L312S mutations were shown to change conformation upon AVP stimulus: not only cAMP generation was further increased but agonist binding of the receptor was also followed by increased β-arrestin2 binding and internalization (42–44). In contrast, R137C/L mutants reflected another conformation: enhanced basal β-arrestin2 binding leads to increased internalization of the mutant receptor and to a much lesser degree, β-arrestin1 binding showed similar characteristics (39, 41).

These results suggest an interesting hypothesis: R137C/L mutations rather than any other NSIAD mutations have unique properties. Mutation of that arginine residue results in ligand insensitivity (and this applies also to the XNDI causing R137H mutation). The experimental data by Vezzi et al. suggest that these mutations create a ‘frozen’ conformation leaving the receptor in a sort of weakly active state (41). In contrast, other NSIAD mutations make more conformations possible. Given the small variety of known NSIAD causing mutations, general statements cannot be identified for gain-of-function mutant V2Rs; every new mutation should be characterized to draw conclusion about the functionality of the receptor.

## 4 Clinical manifestation of V2R mutations

### 4.1 Clinical manifestation and prognosis of XNDI

The diagnostic steps of NDI reflect the main symptoms of the disease. The classic triad is polyuria, hyposthenuria and consequential polydipsia. Patients presenting with polyuria (more than 3 L/day in adults and 2 L/m^2^/day in children), hyposthenuria and polydipsia should be investigated with a water-deprivation test. The lack of urine concentration (reaching urine osmolality of 600 mOsm/kg water at plasma osmolality of 295-300 mOsm/l and/or serumNa^+^ concentration of 145 mM) excludes primary polydipsia. Administration of desmopressin (dDAVP), a potent and selective V2R agonist peptide, helps to differentiate between central diabetes insipidus (impaired AVP secretion) and nephrogenic diabetes insipidus. As the molecular biological characteristics of mutant V2Rs suggest, dDAVP administration does not alter significantly urine osmolality in NDI. In order to avoid dangerous dehydration in small children, only dDAVP may be administered without water deprivation, which proves NDI along with other laboratory tests (SeNa^+^, urine osmolality, serum AVP concentration) (8).

The incidence of congenital XNDI is estimated at 1:100000 birth. Nearly 60% of congenital NDI patients are diagnosed within the first year of life and according to the latest European study, only 6% of NDI cases are diagnosed in adulthood. The late diagnosis showed association with the economic development of the country, but no data are available about the proportion of partial forms of NDI (45). The symptoms of NDI are often frightening in infants, forcing the parents to seek medical help when they observe polyuria. The polyuria may be greatly excessive, which can be as high as 1000 ml/day in a 5 kg infant or 6000 ml/day in a 10 kg, 3-years old child (31, 46). In infants, constant thirst can lead to sucking large amounts resulting in consequential gastro-esophageal reflux and vomiting (11). Decreased height development is one of the earliest observed effects in children, but interestingly, despite of that, the latest report on a large European cohort (relative to the incidence of the disease) showed similar weight development to that of the healthy population, however there are cases presenting decreased weight of NDI children (45, 46).

In adults, the polyuria and polydipsia often exceed 10 L/day. As mentioned earlier, most of the affected and therefore diagnosed patients are males (84% in the large cohort) (45). Variable penetrance, which presents the phenotypic consequence of a mutation in females, can result in various levels of symptoms due to unbiased-skewed X-inactivation. Most female carriers are asymptomatic, while others show mild to severe (similar to male patients) AVP resistance and polyuria, depending on the expression profile of the wild type V2R. These symptoms alone limit the achievable quality of life and create the need for precise everyday life management. The nocturia and the frequent midnight water uptake can lead to poor sleep quality. Despite the decreased height in childhood, the distribution of height does not differ from the healthy population in adulthood. Surprisingly, adult NDI patients showed significant overweight and obesity compared to the general population in European countries (45). It would be interesting to investigate the incidence of obstructive sleep apnea syndrome and obesity-hypoventilation syndrome in NDI patients in the future, considering the coincidence of the increased rate of obesity and sleep impairments.

Long-term effects of congenital NDI consist of several medical issues. It has been described that these adult patients have an increased risk of flow uropathy and related diseases, such as hydronephrosis and bladder dysfunction (11, 45). Chronic kidney disease with deteriorating renal function is more prevalent compared to the general population, especially in older NDI patients (45). Theoretically, the risk of intellectual disability should not be increased with proper healthcare systems, but data suggests that the incidence of mental health problems in adults and attention-deficit hyperactivity disorder in children is higher (45, 47). Although a smaller proportion of NDI patients have achieved academic degrees, their employment rate is similar to the general population (45). Despite these data, it is logical that these patients do not have the same opportunities in the labor market as healthy people, since some working conditions are unsuitable for their condition.

The coincidence of other acute- and chronic illnesses creates another interesting aspect of the clinical course of congenital NDI. The normally fragile balance in water and electrolyte homeostasis could create challenging situations in otherwise life-threatening, critical conditions. Illnesses, injuries and operations causing large fluid shifts (bleeding, extravasation, etc.) may have devastating effects without proper renal compensatory mechanism.

### 4.2 Clinical manifestation and prognosis of NSIAD

The identification of the first known V2R mutations leading to NSIAD reflects the spectrum of symptoms of the disease. Since the neurological symptoms depend on the chronic or acute-on-chronic severity of the hyponatremia, there are large variations in clinical manifestation. It has to be noted that due to the escape mechanisms of water balance regulation (local and endocrine effects), the severity of symptoms is affected by the electrolyte imbalance, and not the total body water, since these patients are euvolemic in the chronic state (48). To date, the gold standard for the diagnosis of NSIAD is the sequencing of the *AVPR2* gene in patients with otherwise unexplainable euvolemic hyponatremia. Indirect proof may be a water challenge proposed by Ranchin et al., where the administration of 10 or 20 ml/kg water to hemi- or heterozygous patients, respectively, results in hyponatremia and induces limited diuresis and concentrated urine (49). AVP concentrations in serum depend on the actual serum sodium concentration and volume state; at symptomatic hyponatremic states the AVP concentration was low or below detection limits in the literature (7, 50).

Reflecting the genetic background, symptomatic NSIAD has been diagnosed in male patients. As stated above, a water challenge (and extrapolating, certain acute diseases resulting in water homeostasis imbalances) may induce hyponatremia in heterozygous females too (7, 42–44, 49, 51). The clinical presentation of the disease dependent on the time of onset and severity of symptoms. A proportion of NSIAD patients do not show any clinical symptoms at the diagnosis and the recognition of the hyponatremia is merely accidental. There are examples in infants and adults, yet, most of the newly diagnosed patients in the literature are infants with symptoms (43, 51). Either acute or acute-on-chronic hyponatremia leads to neurological symptoms, irritability, apnea, and seizures (7, 42). Familial analysis showed that the recurrent onset of seizures may lead to permanent neurological impairment and intellectual disability in NSIAD patients (49).

Although we have very limited information, one of the most interesting questions is how NSIAD disease influences the quality of life and lifetime expectancy of these patients. There are certain conditions that can severely deteriorate NSIAD patients, especially those whose formerly asymptomatic disease is unrecognized. Exercise-induced hyponatremia can affect these patients, but the challenge will be the handling of the coexisting chronic illnesses in the future. Any disease affecting sodium and water balance may complicate NSIAD in elderly patients: chronic renal insufficiency, diseases causing hypovolemic hyponatremia, such as heart failure or hepatic cirrhosis may lead to management difficulties. Not to mention critical illnesses, which very often lead to hyponatremia. It has been shown that the prevalence may reach 40% in the intensive care unit and 14% of patients have low serum sodium concentration at admission due to previously mentioned etiology along with acute deterioration such as hypovolemic hyponatremia (52).

## 5 Causative therapy of V2R mutations

For XNDI, everyday life management may help maintain the normal electrolyte and water balance, however, it is restricted with compromises. Acute disease can deteriorate the condition of these patients quickly, supportive therapy - with limited effectiveness - is the only possible solution at present. In many cases, XNDI patients do not respond to supportive therapy with decreased urine output, even though the chronic sodium concentration and euvolemic state may be achieved (53–55). For symptomatic NSIAD patients, supportive therapy includes fluid restriction, oral urea supplementation and the general avoidance of all possible circumstances leading to rapid water intake (i.e. sport activities, heat waves) (56). Taken together, clinical manifestations of XNDI and NSIAD along with their deleterious effect as complications in other diseases create the absolute need for causative and effective therapy.

### 5.1 Functional characterization methods of V2R mutations

Causative therapy should be based on the cellular consequences of loss- and gain-of-function V2R mutations. As previously presented, XNDI mutations can be classified into well-defined groups according to molecular properties. The known NSIAD mutations can be divided into two groups functionally (depending on inverse agonist sensitivity) with similar properties. Identification of each *AVPR2* mutation should be followed by the characterization of the disease-causing V2R mutant. Although the fast-evolving computational techniques are promising for modeling the cellular consequences of the receptor mutation in the future, the recent ‘gold standard’ methods are molecular biological and biochemical experiments (33). Using heterologous expression systems, the signaling capability, cellular localization, ligand binding, and maturation properties of the mutant V2Rs can be characterized. Moreover, the potential functional rescue effects of different compounds can be tested easily in these experimental setup.

There are numerous possibilities to characterize the biochemical consequences of a mutation in the V2R (either loss-of-function or gain-of-function). However, in order to identify the possible therapeutic strategy, crucial steps include the determination of the plasma membrane expression, AVP binding profiles and the measurement of the intracellular cAMP response. The signaling capability of the mutant receptor should be assessed in response to agonist (mainly AVP) stimulation, and/or inverse agonists treatment. The dose-dependent cAMP production of the mutant receptors could be compared with the agonist-induced wild-type V2R cAMP production. Investigating the internalization properties may provide further information about the regulation of the mutant receptor. In addition to the short-term effects of AVP induced V2R activation, it can be important to investigate the long-term effects, such as CREB phosphorylation or AQP2 expression levels. As a typical translational field between basic science and clinical investigation, all of the techniques used for functional characterization should be well-established, widely accepted and standardized for better comparison in the future. It is also important to highlight that

The measurement of the plasma membrane expression of mutant V2R has several reliable, sensitive methods in the literature. Fluorescence protein-tagged receptors or epitopes containing receptors tagged with fluorescent antibodies can be investigated with laser scanning microscopic technique and quantified using software-based methods (57, 58). Moreover, using permeabilized conditions during the immunodetection could reveal the magnitude of the intracellularly located receptors in case of intracellular trapping or impaired trafficking due to the mutation in the receptor molecule. The total cellular expression of the receptors can also be determined in western-blot experiments using the receptor expressing cell lysates; this method also can reveal the molecular weight and the glycosylation pattern of the mutant receptors (59). HA-epitope containing cell surface receptors can also be quantified using flow cytometry (43). The BRET (bioluminescence resonance energy transfer)-based method can be used since it is highly sensitive and can also monitor dynamic changes in the distance between the tagged receptor and a plasma membrane anchored protein (43, 54).

There are numerous methods used for cAMP signal generation capability in the investigation of both loss-of-function and gain-of-function mutations of the V2R. Techniques of cAMP detection kits, bioluminescence-based reporter gene assays are capable of measuring the cAMP signal at a definitive moment (34, 39, 60). Other methods are more suitable for dynamic monitoring of the second messenger generation, such as the Epac-based BRET biosensor, which also provides information about the signal kinetics (54). The BRET method can also be used for the measurement of receptor trafficking and β-arrestin-binding properties of the mutant receptors (54, 59, 61). In some cases, it is necessary to determine the overall effect of V2R stimulation on AQP2 expression. Previous studies have reported that the expression level of AQP2 depends on Epac activation and CREB (25, 62). The presence of AQP2 or CREB phosphorylation can be checked using specific antibodies in imaging or western-blot determinations, but the AQP2 expression can also be investigated by AQP2 promoter-luciferase reporter assay, as well (25).

The determination of AVP binding properties of mutant V2Rs is also important in the characterization of the impaired function, especially in situations where the cell surface expression of the mutant V2R is normal but the AVP hormone induced signaling is severely impaired. AVP saturation binding assays using [^3^H]AVP as a radioligand can be useful tool to determine the binding affinity of the mutant receptors compared to the wild-type V2R. This method can also be used to measure the binding affinity of various compounds to the mutant V2Rs in [^3^H]AVP displacement binding assays (59).

The methods mentioned above can serve valuable data on V2R functionality in heterologous expression systems. However, any therapeutic strategies based on these *in vitro* results should be further confirmed by additional *in vivo* studies. For instance, a compound that appears to successfully restore impaired mutant V2R functions in heterologous expression may not be enough to induce urine concentration in animal studies or clinical tests. Therefore, mutational and *in vitro* analyses are necessary for determining the class of V2R mutations and suggesting potential treatment possibilities, but animal models and clinical trials should be used to evaluate the effectiveness and safety of the proposed compounds (63).

### 5.2 Therapeutic strategies for XNDI

As previously presented, different classes of V2R mutations can cause XNDI. Class I mutations result in either largely defective receptor synthesis or no receptor expression at all, leading to non-salvageable cellular consequences. Class II mutations, which are responsible for the significant proportion of the XNDI causing V2R mutations, result in ER retention of the misfolded receptor molecules. Similar to other GPCRs, the rescue of these mutant receptors with pharmacochaperones was a major breakthrough. Since approximately 50% of all NDI mutations are missense, in which a pharmacological chaperone-based therapy may represent a possible general treatment for this protein-misfolding disease (63, 64). One of the main limitations of pharmacochaperones is that their effects are significantly dependent on the given V2R mutation, thus different mutations may require unique pharmacochaperones to achieve functional rescue of the mutant V2R receptor. The other potential problem can be the consequence of the non-complete selectivity of pharmacochaperones to the V2R, leading to unwanted activation or inhibition of other receptors. The nonpeptide, cell permeable inverse agonists have been shown to bind to misfolded V2Rs in the ER, creating an antagonist-receptor complex, which can bypass the ER quality control system (63, 65, 66). Pharmacochaperones can alter the configuration of the misfolded mutant receptor molecules thus allowing the transport of intracellularly trapped receptors to the plasma membrane, where these rescued receptors can bind AVP (58, 63, 65). In the earliest investigations, these pharmacochaperones were inverse agonists. Although tolvaptan (OPC41061), a nonpeptide V2R selective inverse agonist, has the advantage that it is already an approved drug (Samsca) for medical use for specific indications such as heart failure, polycystic kidney disease (with a warning for potential hepatotoxicity), its effectivity in XNDI is uncertain (67). The rescue of the mutant V2R is rather in vain if the persisting presence of the inverse agonist blocks the receptor function. The terminal half-life of tolvaptan is relatively long, according to studies varying between 9-12 hours. Despite that, theoretically it is possible that with a fluctuant tolvaptan plasma concentration a certain, functioning phase may be reached with the rescued, antagonist-unbounded mutant V2R, especially in those XNDI patients who live without any V2R function, which cannot be further blocked with the inverse agonist. Another intriguing molecule, the SR49059 (relcovaptan) has been shown to achieve receptor rescue not just in cellular models, but also decreased urine output in XNDI patients (57, 58, 63). However, the future clinical use of SR49059 is ambiguous, since the previous XNDI trial was terminated because of cytochrome P450 interference (63, 68). It has to be mentioned, that this compound is a V1aR selective antagonist (affinity is two orders of magnitude higher than to V2R), limiting the use of SR49059 in clinical situations when there is an absolute need for decreased polyuria in XNDI patients together with the lifesaving compensatory effect of AVP on other receptor subtypes (e.g. septic shock, bleeding of an XNDI patient) (69). Pharmacochaperone agonists would possess advantages over antagonists since their capability to directly stimulate mutant and rescued V2R molecules and they can induce second messenger signaling and AQP2 translocation into the luminal membrane of principal cells. Nonpeptide, cell permeable V2R agonists are in the center of interest in the recent years. As agonist ligands, VA88, VA89, MCF14, MCF18 and MCF58 have shown to rescue misfolded receptors and restore cAMP signaling (31, 59, 70). Interestingly, the VA88 and VA89 compounds showed intracellular activation of mutant receptors, whereas the MCF14 compound can also promote the maturation of some V2R mutant receptors. It has also been reported that MCF-induced receptor activation did not initiate β-arrestin binding. The lack of induced internalization and downregulation may be therapeutically beneficial for signal- selective biased agonists (59). Although these compounds have great potential in the treatment of XNDI, unfortunately, neither of these compounds were administered to humans as part of clinical studies.

A new therapeutic approach may be considered in the case of class III V2R mutations. It was demonstrated that impaired G protein coupling leads to altered agonist sensitivity of the V2R, and examination of the dose-response curve showed that the mutation affects the potency of V2R selective peptide agonists differently (54). In that particular case, Val4-desmopressin had a distinctively higher potency than other peptide agonists. Theoretically, with a similar measurement setup, an optimal peptide agonist may be found for class III mutations, including mutations leading to a decrease in affinity to natural ligand. A few peptide V2R agonists, other than desmopressin, have been already used in clinical studies in the past (71).

### 5.3 Therapeutic strategies for NSIAD

In certain clinical situations, as previously discussed), even the otherwise asymptomatic NSIAD patients may benefit from causal therapy. The basis of targeted therapy is the inverse agonist tolvaptan, since this compound (in contrast to other inverse agonists, such as satavaptan) has already been approved as a therapeutical agent for specific indications. The investigation of the effect of tolvaptan on the mutant V2R ensures individualized therapy and the highest risk-benefit ratio. Although only the mutations of the R137 residue were shown to lead to tolvaptan-insensitive V2R, identification of new mutations with similar molecular properties should not be ruled out (48). Close monitoring of hepatotoxic side effects of tolvaptan may provide a safe clinical setting for adults and there are presented studies for pediatric use in certain indications (72, 73). Taken together, the therapeutic use of tolvaptan in NSIAD patient may be achieved in the immediate future.

## 6 Conclusion

Currently, there is no specific treatment is available to restore the function of the mutant V2R to treat patients with XNDI or NSIAD. The cellular consequences of V2R mutations form the pathophysiological background of a wide spectrum of clinical symptoms in XNDI and NSIAD patients. Functional characterization of these mutations creates the opportunity of causal therapy for these diseases. Targeted therapy may be the solution not only for better quality of life and long-term prognosis but also for the concomitance of chronic diseases or acute deteriorations. These rare diseases affect a small proportion of the population, creating the absolute need for academic research as the basis of new therapeutic strategies.

## Author contributions

All listed authors have made a substantial, direct, and intellectual contribution to the work and have approved it for publication.
